# Isoliquiritigenin modulates miR-374a/PTEN/Akt axis to suppress breast cancer tumorigenesis and metastasis

**DOI:** 10.1038/s41598-017-08422-y

**Published:** 2017-08-21

**Authors:** Fu Peng, Hailin Tang, Peng Liu, Jiangang Shen, Xinyuan Guan, Xiaofang Xie, Jihai Gao, Liang Xiong, Lei Jia, Jianping Chen, Cheng Peng

**Affiliations:** 1 School of Chinese Medicine, The University of Hong Kong, Pokfulam, Hong Kong; 2 Chengdu University of Traditional Chinese Medicine, Chengdu, China; 3 State Key Laboratory Breeding Base of Systematic Research, Development and Utilization of Chinese Medicine Resources, Sichuan Province and Ministry of Science and Technology, Chengdu, China; 4 Department of Breast Oncology, Sun Yat-sen University Cancer Center; State Key Laboratory of Oncology in South China; Collaborative Innovation Center of Cancer Medicine, Guangzhou, China; 5 Department of Clinical Oncology, Li Ka Shing Faculty of Medicine, the University of Hong Kong, Pokfulam, Hong Kong

**Keywords:** Breast cancer, Apoptosis, Cell invasion, Drug development

## Abstract

Breast cancer is one of the most frightful causes of death among females worldwide. Accumulating evidence attached the importance of microRNAs negative regulation to tumorigenesis in breast cancer, suggesting novel cancer therapies targeting microRNAs modulation. Recent studies demonstrated that isoliquiritigenin could inhibit breast cancer cells proliferation and migration, but the underlying mechanism is still limited. In this study, the anti-cancer effects as well as the detailed mechanisms of isoliquiritigenin were explored. The results proved that isoliquiritigenin could negatively regulate breast cancer growth through the induction of apoptosis. We also verified the anti-cancer effect of isoliquiritigenin on migration and invasion, and identified highly expressed miR-374a as one of the main microRNAs down-regulated by isoliquiritigenin treatment in breast cancer. Further study displayed that isoliquiritigenin increased PTEN expression through the decrease of miR-374a expression to inhibit the aberrant Akt signaling. Our findings suggest isoliquiritigenin as a novel anti-cancer candidate significantly regulating miR-374a/PTEN/Akt axis in microRNA-based breast cancer therapies.

## Introduction

Breast cancer, especially triple negative breast cancer (TNBC), is considered as one of the most aggressive cancers and one of the major causes of mortality among female worldwide. Although the early-detection system and multidisciplinary treatments have improved, breast cancer incidences and recurrence ratios remain unsatisfactory, especially for developed countries^1^. In 2015, it was reported by the Breast Cancer Research Foundation that emerging 231,840 American women had been diagnosed with invasive breast cancer^2^. Hence, developing novel biomarkers and approaches focusing on breast cancer prevention and treatment has become an urgent issue.

In the recent decades, accumulating evidences indicated that small non-coding RNA molecules (*i.e*., miRNAs), as negative gene regulators with the ability to induce targeted mRNAs degradation and/or translational inhibition, could govern cell proliferation, cell migration, cell invasion, cell differentiation and angiogenesis in the development and progression of breast cancer^3–7^. Recently, miRNA profiling demonstrated that miR-374a was highly overexpressed in several different types of human cancer, including head and neck cancer, follicular lymphoma, and small cell lung cancer^8–10^. In esophageal cancer, high expression of miR-374a could promote proliferation via directly targeting Axin2, an inducer of apoptosis^11^. In breast cancer, miR-374a was remarkably upregulated in metastatic breast cancer cells through the direct target of WIF1, Wnt5a, and PTEN, which is a key suppressor of oncogenic PI3K/Akt signaling in breast cancer, especially in TNBCs, and positively correlated to disease-free survival ratio and negatively correlated to invasive grade^12, 13^. Interestingly, higher expression of miR-374a was correlated with better prognosis among TNBC patients^14^. However, whether miR-374a could promote breast cancer proliferation is still unclear.

Since miRNAs, with capacity of single miRNA interacting with multiple mRNAs, are the most substantial regulatory genes on the gene expression network, reports in increasing number initially concentrate on developing miRNA-based strategies for cancer therapies^15–17^. Well-known tumor suppressor miRNAs have been gradually assessed in clinical trials is of benefit to cancer patients. For an instance, miR MRX34 used in a phase-I clinical trial (NCT01829971) for liver cancer therapy is underway^18^. Recently, attention had shifted towards traditional Chinese medicine (TCM) for the treatment of multiple malignancy-related processes because of the low toxicity and high tolerability, and single compounds from TCM are gaining acceptance as potently potential anticancer agents targeting oncogenic miRNAs (*e.g*., berberine, curcumin, and resveratrol)^19–21^. Isoliquiritigenin (ISL), a natural flavonoid majorly derived from the root of licorice, possesses anti-cancer activities at multistage carcinogenesis processes, including proliferation suppression, cell cycle arrest, angiogenesis inhibition, metastasis obstruction and apoptosis induction in various types of cancer^22–24^. In our pervious work, ISL showed an essential inhibitory effect on breast cancer through the inhibition of angiogenesis, the activation of autophagy, and the repression of breast cancer stem cells with minimal effects on the proliferation of the MCF-10A normal human mammary epithelial cell line and normal tissues^25–28^. Additionally, its inhibitory effect on breast cancer growth and migration has been reported currently. ISL induced growth suppression and apoptosis *in vitro* and *in vivo* via repressing Arachidonic acid metabolic network and inactivating Akt pathway^29^. Its anti-migratory effect was confirmed in MDA-MB-231 cells through the reduction of VEGF secretion and the inhibition of PI3K/Akt expression^30^. Although ISL limited breast cancer proliferative and migratory ability, its suppressive effect on breast cancer metastasis and underlying mechanisms on PTEN/Akt signaling deserve further investigation.

In the present study, we addressed the role of ISL on mitochondria-based apoptosis induction and metastasis suppression of breast cancer *in vitro* and *in vivo*. Microarray analysis further revealed that miR-374a was one of the primary targets of ISL and negatively correlated to the pro-apoptotic effect and anti-metastatic effect of ISL. Notably, ISL-induced apoptosis and metastatic suppression was partly dependent on the increase of PTEN, which was the direct target of miR-374a ensured by luciferase assay. Taken together, our results unearthed a novel function of ISL as a natural miR-374a inhibitor to suppress breast cancer tumorigenesis and metastasis by regulating the aberrant Akt signaling.

## Results

### ISL inhibits cell proliferation and induces apoptosis of breast cancer cells

**Figure 1:**
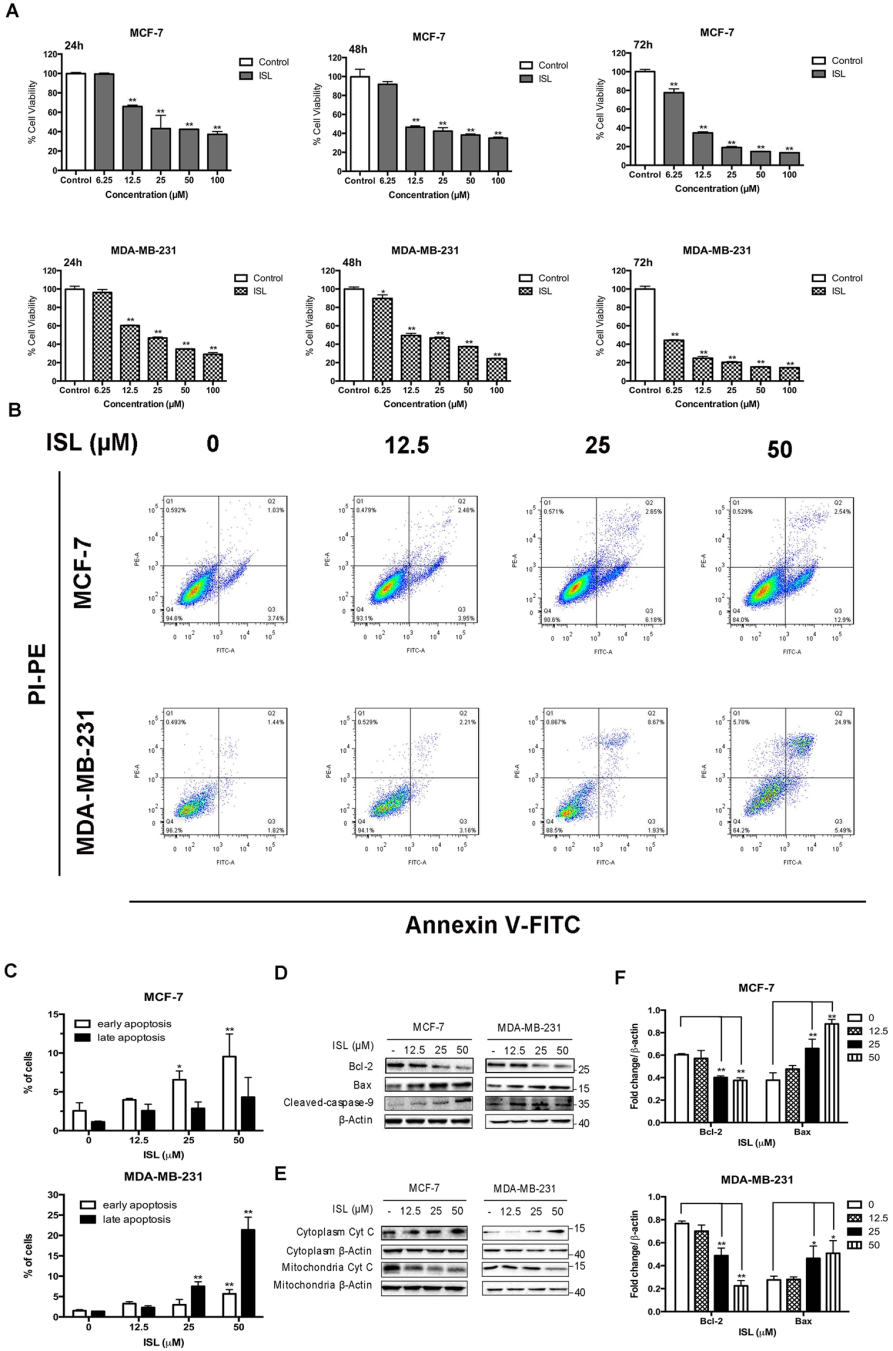
Effect of ISL on cell proliferation and apoptosis in breast cancer cells. (**A**) Cell viability of MCF-7 and MDA-MB-231 after 24 h, 48 h, 72 h ISL treatment. (**B**) Representative images of induction of apoptosis in breast cancer cells determined by Flow cytometry analysis cultured with ISL (12.5, 25, 50 μM) for 24 h. (**C**) Percentages of early apoptotic cells and late apoptotic cells in different dose of ISL intervention according to Flow cytometry assay analysis. (**D**,**F**) Western blot analysis of Bcl-2, Bax, Cleaved Caspase-9, in MCF-7 and MDA-MB-231 after 24 h ISL treatment. The full-length blots were presented in the Supplementary Fig. 7. (**E**) Western blot analysis of Cyt C release in breast cancer cells after 24 h ISL interference. The full-length blots were presented in the Supplementary Fig. 8. Data represent the mean ± s.d. *P < 0.05, **P < 0.01.

To determine whether ISL suppressed breast cancer cells, we tested the effects of ISL on the growth of human breast carcinoma cells using MTT assay. After ISL 24 h, 48 h, 72 h treatment, the cell viability was assessed. ISL treatment resulted in a dose-dependent inhibition of cell viability, with IC_50_ of 32.66 μM and 22.36 μM for 24 h treatment on MCF-7 and MDA-MB-231, respectively. At the dose of 6.25 μM, ISL did not affect the viability of MCF-7 and MDA-MB-231 cells. ISL at 25 μM dramatically inhibited the breast cancer cells growth and induced a time-dependent decrease of cell numbers (*P* = 0.002, and *P* < 0.0001, respectively) (Fig. 1A). Further study demonstrated that ISL also displayed an inhibitory effect on primary culture of breast cancer proliferation (see Supplementary Fig. S1). The results from flow cytometry (*i.e*, Annexin V-FITC and propidium iodide (PI) staining) determined that ISL at low concentration did not manifest significant effect on apoptosis in breast cancer cells, while the effect of ISL at 25 μM and 50 μM was, at least mostly, through inducing apoptosis (For early apoptosis, *P* = 0.0038, and *P* = 0.0031, respectively, and for late apoptosis, *P* = 0.127, and *P* < 0.0001, respectively with ANOVA test) (Fig. 1B,C). 24 h ISL treatment significantly decreased Bcl-2 expression and increased Bax expression in a dose-dependent manner of in a range from 6.25 to 50 μM (For Bcl-2, *P* = 0.0302 and *P* = 0.0032, respectively, and for Bax, *P* = 0.003 and *P* = 0.0471, respectively with ANOVA test). We also measured cleavage of caspase-9 and the release of cytochrome c (Cyt C) from the mitochondria. The content of cytoplasmic Cyt C and cleaved caspase-9 were dose-dependently increased in MCF-7 and MDA-MB-231 cells by ISL treatment for 24 h (Fig. 1D,E). Thus, we chose ISL for further mechanism exploration of cell motility study at 6.25 μM and cell apoptosis study at 25 μM.

### ISL suppresses breast cancer growth and lung metastasis *in vivo*

**Figure 2:**
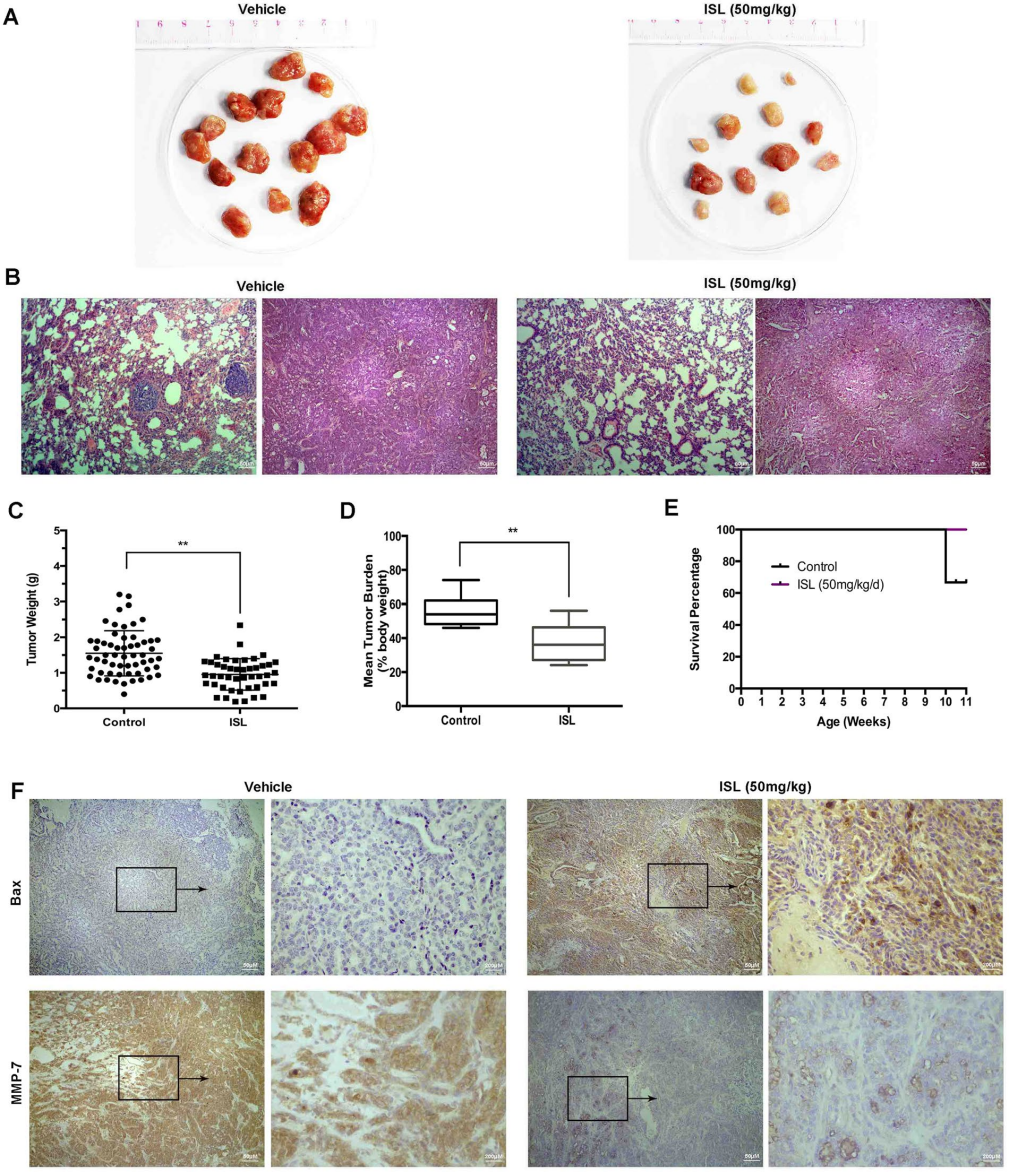
ISL inhibits tumorigenesis and metastasis in MMTV-PyMT transgenic mice. (**A**) Representative images of solid tumors collected from vehicle group and ISL treatment group (50 mg/kg/d). (**B**) Representative micromorphology of the dissected tumors and lungs with HE staining in 100-fold magnification. (**C**) Scatter plots of individual tumors with mean weights at the end point of ISL treatment (n = 6). (**D**) Data about tumor burden of mice from vehicle and ISL-treated (n = 6). (**E**) Kaplan-Meier curve of mice survival in two different groups after ISL administration (n = 6). (**F**) IHC staining analysis of Bax and MMP-7 in vehicle tumor tissues and ISL-treated tumor tissues. Data represent the mean ± s.d. **P < 0.01.

We used MMTV-PyMT mice model to further elucidate the *in vivo* anti-cancer effect of ISL on breast cancer growth and metastasis. Since it has been acknowledged that MMTV-PyMT female mice could develop palpable luminal-type mammary tumors metastasizing to the lung in or after the 10^th^ week^31, 32^, ISL (50 mg/kg/d) were administered to the mice at 4^th^ week from birth through oral intake, and at the 11^th^ week of ISL treatment, mice had been sacrificed and tumors had been dissected from mice. Mammary tumors in vehicle group demonstrated a more hemorrhagic appearance than ISL treatment group, while the average size of ISL-treated tumors was dramatically smaller than that of vehicle group (Fig. 2A). Further histopathologic analysis indicated that both groups displayed typical features of malignancy, although less metastatic nodules were visible in ISL-treated group (Fig. 2B). The mean of tumor weight and tumor burden were considerably reduced after ISL administration for 11 weeks (Fig. 2C,D), and the survival span and ratio of MMTV-PyMT mice were prolonged with ISL treatment (Fig. 2E). No significant change of morphology was found out on normal tissues treated with ISL (see Supplementary Fig. S2). MMP7 is a matrix metallopeptidase involved in invasion and metastasis in multiple cancers including breast cancer^33–35^. Further IHC staining using anti-Bax and anti-MMP-7 revealed that the expression of Bax was substantially increased with ISL treatment, and MMP-7 expression was remarkably decreased, respectively (Fig. 2F). Collectively, these data manifest that ISL has a remarkable inhibitory effect on breast cancer tumorigenesis and pulmonary metastasis.

### ISL reduces overexpressed miR-374a in breast cancer cells

**Figure 3:**
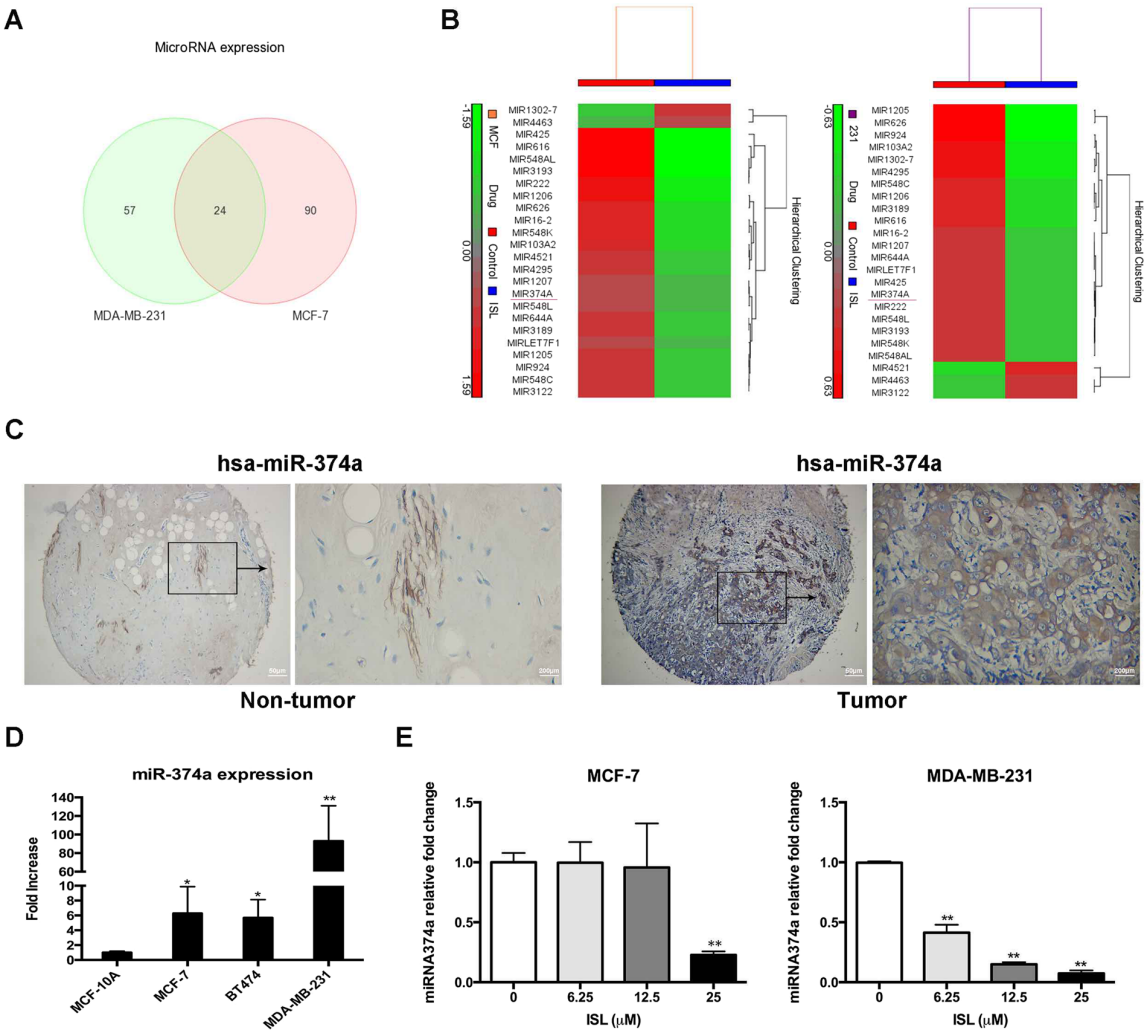
MiR-374a is highly expressed and attenuated by ISL in breast cancer. (**A**,**B**) Microarray analysis of miRNAs differences after 24 h ISL treatment in MCF-7 and MDA-MB-231. (**C**) ISH analysis of miR-374a expression in tumor tissues and matched normal tissues from breast cancer patients. (**D**) qRT-PCR analysis of miR-374a levels in human cancer cell lines and spontaneously immortalized MCF-10A cells. (**E**) qRT-PCR analysis of differently expressed miR-374a levels at the indicated concentrations of ISL.

Microarray was performed to analyze the miRNA expression at least 1.5 fold change regulated by ISL. 24 miRNAs expression were markedly altered in both MCF-7 and MDA-MB-231 cells cultured with ISL (25 μM) for 24 h (Fig. 3A). Out of the miRNAs screened, 1 miRNA was consistently up-regulated and 20 miRNAs were steadily down-regulated after ISL interference (Fig. 3B). Data downloaded from array express (https://www.ebi.ac.uk/arrayexpress/) and developed in Università di Ferrara demonstrated that miR-374a was reliably remarkably up-regulated in breast cancer patient tissues compared with the marched normal tissues among 20 miRNA differences analyzing with Morpheus (https://software.broadinstitute.org/morpheus/) (see Supplementary Fig. S3). *In situ* hybridization analysis further confirmed the up-regulation of miR-374a in breast cancer patients (Fig. 3C). The expression of miR-374a expression was examined in 39 breast cancer tissue samples, and high level of miR-374a was associated with the clinical stage (*P* = 0.037) (Table 1). Findings from qRT-PCR analysis displayed that miR-374a was highly expressed in breast cancer cell lines and breast, especially for highly metastatic MDA-MB-231 (Fig. 3D, *P* = 0.0323, *P* = 0296, and *P* = 0.001, respectively). Using qRT-PCR, we validated that ISL decreased miR-374a expression in a dose-dependent manner (Fig. 3E), indicating miR-374a was a highly breast cancer-associated oncogenic miRNA modulated by ISL intervention.

**Table 1:** Relationship between miR-374a level and clinicopathologic parameters of breast cancer.

Variable	Number of cases	miR-374a		
		High	Low	*P* value
**Age (years)**				
≥50	18	11	7	0.802
<50	21	12	9	
**Pathologic grade**				
1, 2	25	16	9	0.201
3	14	6	8	
**Clinical stage**				
Ι, ΙΙ	25	11	14	0.037
ΙΙΙ	14	11	3	
**Lymph node status**				
Metastasis	19	12	7	0.408
No metastasis	20	10	10	

### ISL triggers apoptosis through the down-regulation of miR-374a in breast cancer cells

**Figure 4:**
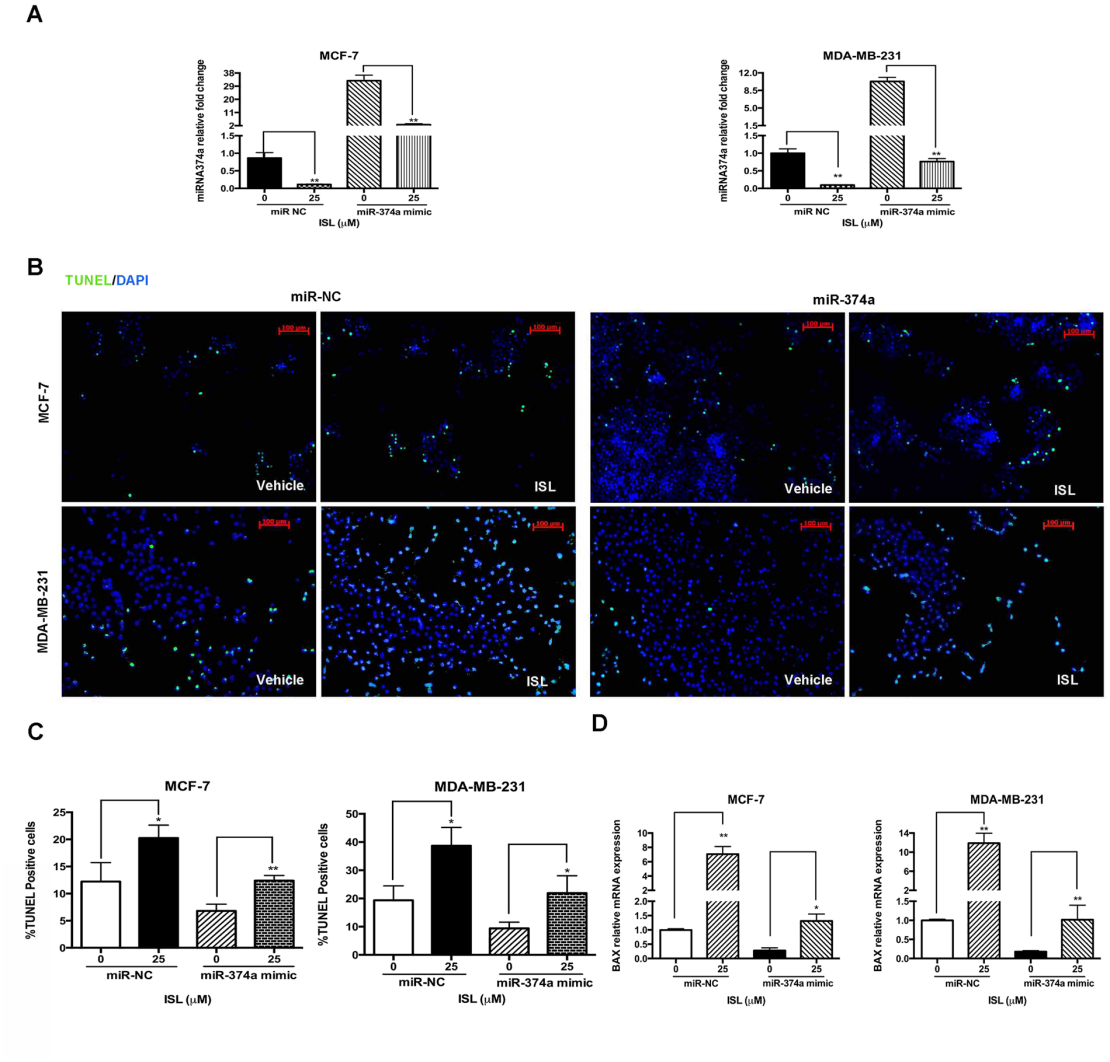
ISL induces apoptosis by regulating miR-374a. (**A**) qRT-PCR analysis of expression levels of miR-374a in miR-NC and miR-374a transfection groups treated with vehicle/ISL for 24 h. (**B**) Representative images of apoptotic cells determined by TUNEL assay in breast cancer cells after 24 h treatment. (**C**) Percentages of TUNEL positive cells in MCF-7 and MDA-MB-231. (**D**) qRT-PCR analysis of the effect of miR-374a interference on BCL-2 and BAX mRNA expression treated with ISL. Data represent the mean ± s.d. *P < 0.05, **P < 0.01.

Using Real-time PCR, we confirmed the transfection of miR-374a into breast cancer cells and the down-regulation by ISL interference (Fig. 4A). Then, apoptotic cells percentages were analyzed through TUNEL staining. The results showed that the suppression of miR-374a by ISL treatment resulted in a significant increase of the number of TUNEL positive cells (*P* = 0.0303, and *P* = 0.0156, respectively), while the overexpression of miR-374a decreased the percentage of TUNEL positive cells (Fig. 4B,C). In addition, PCR results further revealed that miR-374a mimic decreased BAX expression, and increased BCL-2 expression on the mRNA level, while ISL reversed the anti-apoptotic effect of miR-374a in both MCF-7 and MDA-MB-231 cells (Fig. 4D), suggesting that miR-374a plays an essential role in the responses to ISL exposure in breast cancer cells.

### ISL inhibits miR-374a expression and suppresses migration and invasion of MDA-MB-231

**Figure 5:**
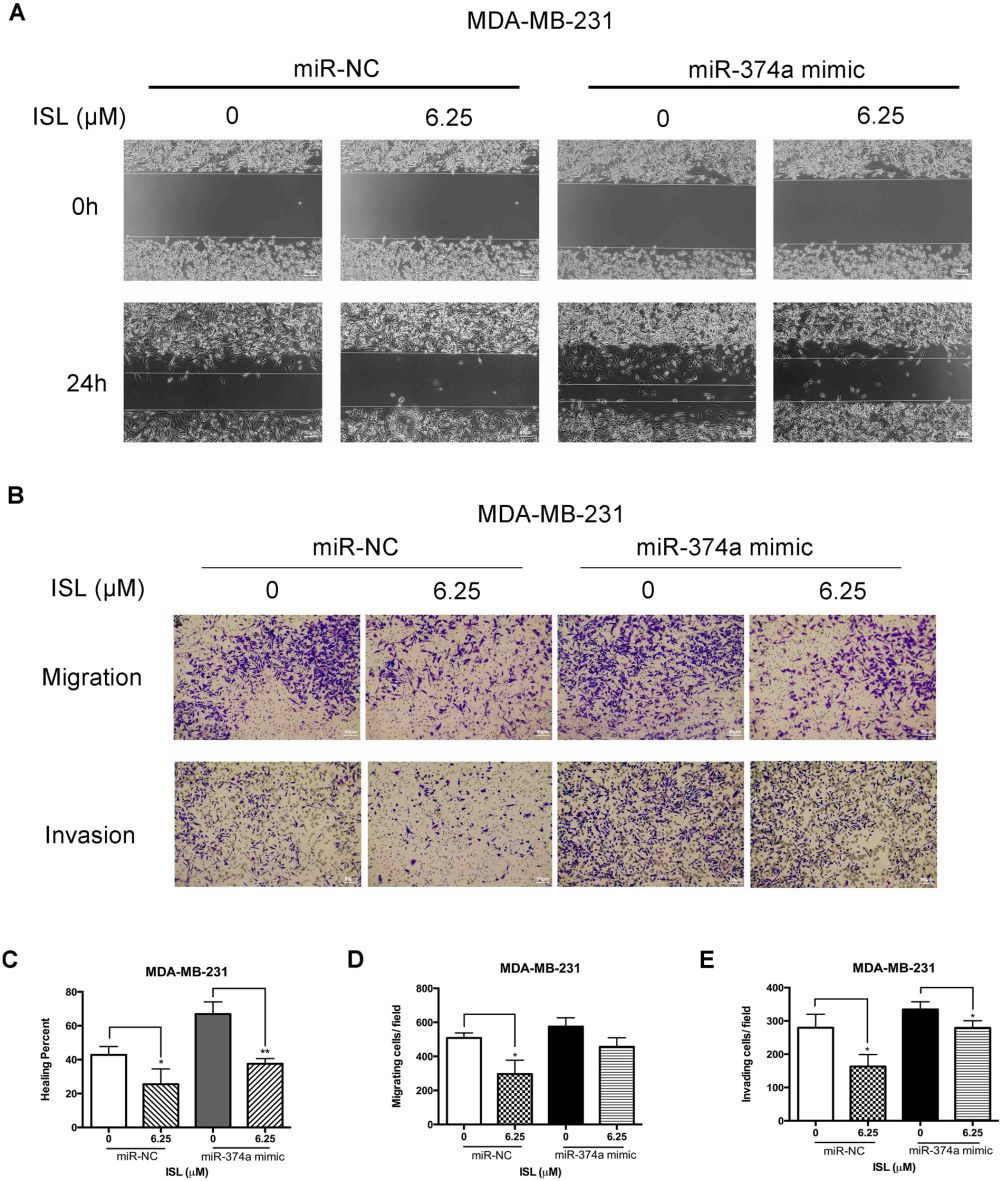
Pretreatment of miR-374a in MDA-MB-231 attenuates the responses to ISL. (**A**) Representative images of wound healing assay in miR-NC and miR-374a mimic transfected groups after 24 h ISL treatment. (**B**) Chamber migration and invasion assay analysis of the effect of miR-374a transfection on breast cancer motile ability with ISL interference. (**C**) Percentages of closures of the wound in miR-374a-modulated MDA-MB-231 cells by 24 h exposure to ISL. (**D**,**E**) Percentages of the number of cells located on the lower side of chambers and Matrigel-coated chambers in the presence of ISL with miR-374a interference. Data represent the mean ± s.d. *P < 0.05, **P < 0.01.

Although ISL displayed a significant anti-migration effect on MDA-MB-231 in the pervious reports^25, 30^, the detailed mechanisms of its effect on anti-migration and anti-invasion are still unclear. The effect of ISL on migration of MDA-MB-231, with or without the miR-374a interference, was determined by wound healing assay, and confirmed by chamber migration assay. After 24 h exposure, ISL-treated cells, whether transfected with miR-374a mimic or miRNA mimic negative control (miR-NC), demonstrated a noticeable delay of responses to moving into wound area, compared with control group (Fig. 5A,C). The results from chamber migration assay showed the inhibitory effect of ISL were at least partly blocked by miR-374a transfection (Fig. 5B,D). Cells invasiveness assessed by transwell coated with Matrigel at 24 h showed similar inhibition by ISL and similarly revealed the interference of miR-374a in the inhibitory effect of ISL on cell invasive ability (Fig. 5C,E). As for MCF-7, ISL did not display the inhibitory effect on migration and invasion at the dose of 6.25 μM (see Supplementary Fig. S4). As for primary culture of breast cancer invasion, ISL showed an obvious inhibitory effect (see Supplementary Fig. S5). Taken together, ISL suppresses the migratory and invasive capacities of MDA-MB-231 through the dramatic suppression of miR-374a.

### ISL regulates miR-374a/PTEN/Akt/β-catenin axis

**Figure 6:**
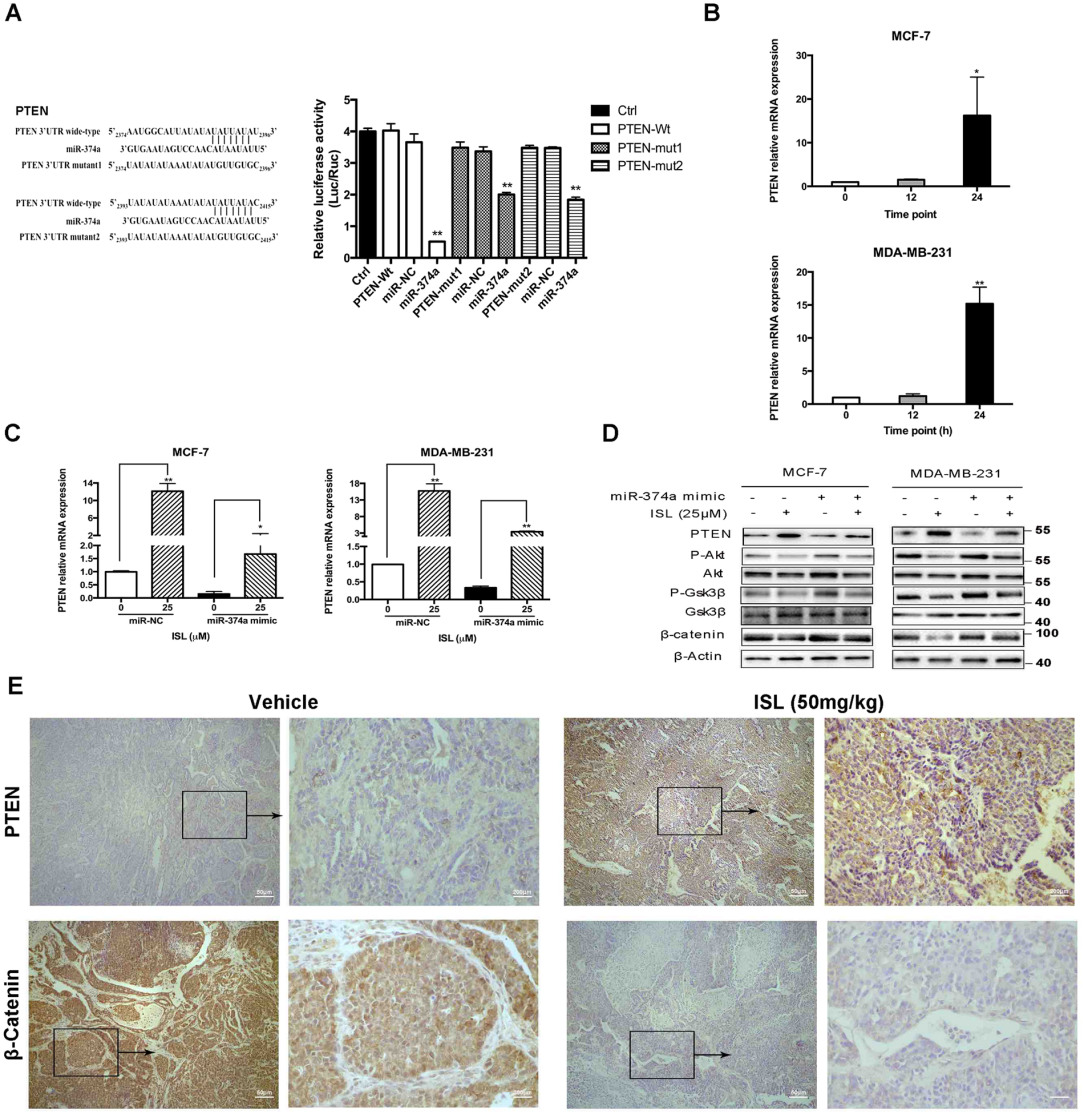
MiR-374a/PTEN/Akt axis is involved in ISL inhibition of breast cancer. (**A**) Dual luciferase reporter assay analysis of the effect of miR-374a expression on 3’UTR of PTEN in 293 T cells. (**B**) qRT-PCR analysis of miR-374a targeted gene expression at the indicated time points in the presence of ISL. (**C**) qRT-PCR analysis of PTEN mRNA expression in normal and miR-374a-overexperssing breast cancer cells interfered with ISL. (**D**) Western blot analysis of miR-374a function on PTEN up-regulation, Akt inhibition, GSK3β suppression, and β-catenin down-regulation by ISL. The full-length blots were presented in the Supplementary Fig. 9. (**E**) Representative images of IHC analysis of PTEN and β-catenin expression in vehicle and ISL-treated tumors.

TargetScan and Microrna.org software predicted one of the main targets of miR-374a was PTEN, the potent inhibitor of Akt pathway. Dual luciferase reporter assay confirmed the direct interaction of miR-374a and its predicted binding sites of 3′UTR region of PTEN (Fig. 6A). To evaluate the effect of miR-374a down-regulated by ISL on PTEN expression, we tested PTEN mRNA expression after ISL intervention. As shown in Fig. 6B, the increase of PTEN mRNA level by ISL was remarkable at 24 h point, and the up-regulated effect was at least partly alleviated by miR-374a mimic pretreatment (Fig. 6C). Western blot analyses confirmed the amplified of PTEN expression by ISL and displayed that ISL inhibited Akt and GSK3β phosphorylation, resulting in a decrease of β-catenin expression. Additionally, ISL-induced Akt/GSK3β/β-catenin inhibition was partly reversed with miR-374a mimic transfection (Fig. 6D). The *in vivo* data were in consistent with *in vitro* results. ISL considerably increased PTEN expression and decreased β-catenin expression determined by IHC staining (Fig. 6E). Altogether, miR-374a is the functionally relevant effector of PTEN/Akt/β-catenin modulation by ISL.

### Modulation of PTEN expression affects ISL-induced apoptosis and invasion inhibition

**Figure 7:**
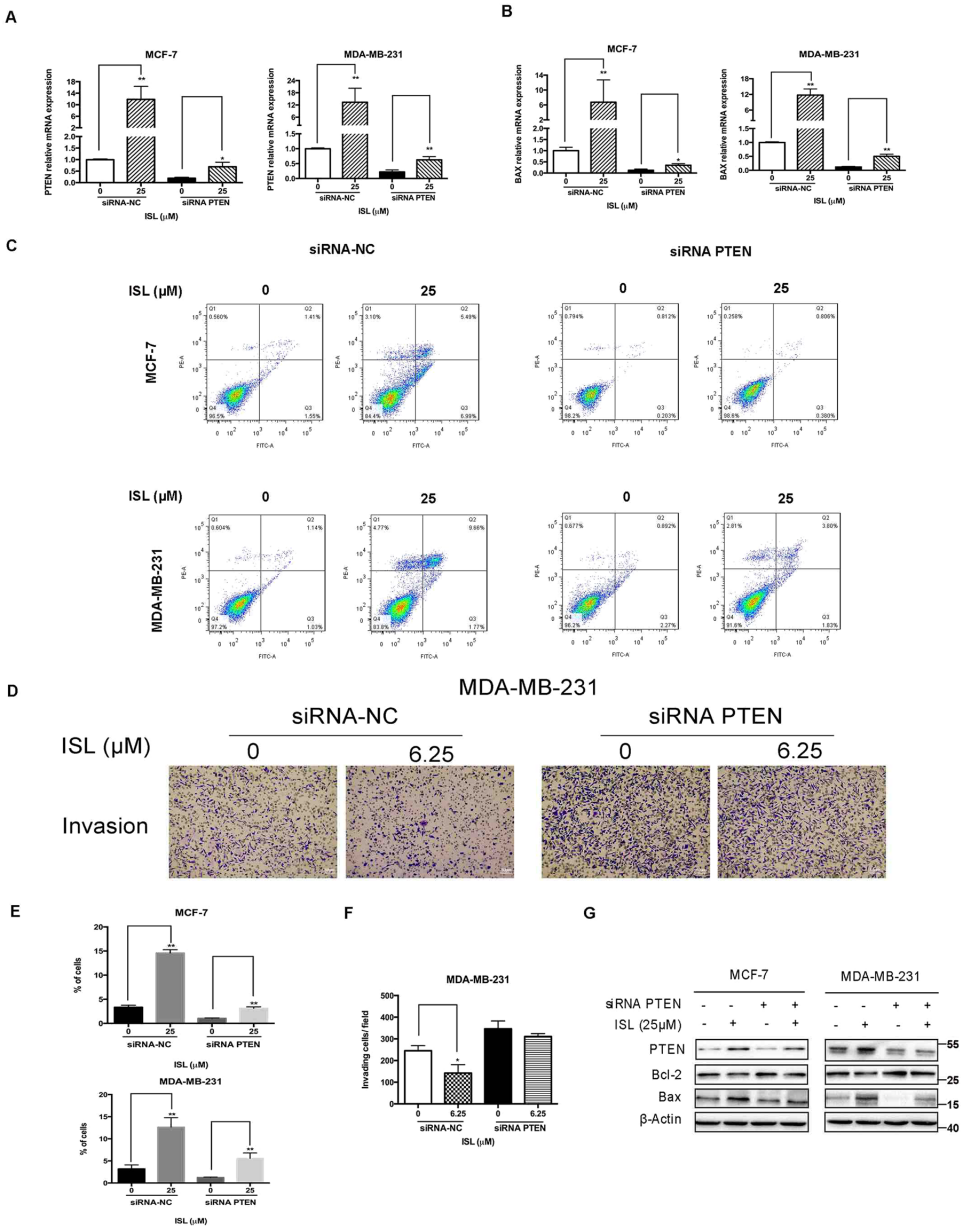
Deletion of PTEN expression restricts the anti-cancer effect of ISL on breast cancer cells. (**A**) qRT-PCR analysis of PTEN mRNA expression in siRNA-NC and siRNA PTEN transfection groups after 24 h ISL treatment. (**B**) qRT-PCR analysis of BAX mRNA expression in siRNA-NC and siRNA PTEN transfection groups with ISL interference. (**C**) Representative images of induction of apoptosis in breast cancer cells determined by Flow cytometry analysis after 24 h ISL intervention. (**D**) Chamber invasion assay analysis of the effect of siRNA PTEN transfection on breast cancer motile ability with ISL treatment for 24 h. (**E**) Percentages of apoptotic cells after ISL treatment with siRNA PTEN intervention. (**F**) Percentages of the number of cells invading in the ISL treatment with siRNA PTEN interference. (**G**) Western blot analysis of PTEN function on Bcl-2 down-regulation and Bax up-regulation by ISL. The full-length blots were presented in the Supplementary Fig. 10. Data represent the mean ± s.d. *P < 0.05, **P < 0.01.

We used qRT-PCR to confirm the down-regulation of PTEN by siRNA PTEN and up-regulation of PTEN by ISL, and siRNA negative control (siRNA-NC) was used as the siRNA transfection control. (Fig. 7A). The results of qRT-PCR analysis also demonstrated that the decrease of PTEN would considerably block the BAX mRNA increase by ISL treatment (Fig. 7B). The pro-apoptotic effect and anti-invasive effect of ISL were also affected after the decrease of PTEN expression (Fig. 7C,D,E,F). Additionally, after transfected with siRNA PTEN, the effect of ISL on biomarkers in apoptosis on protein levels were also partly reversed (Fig. 7G). Collectively, PTEN, the suppressor of Akt pathway, is the essential responder to ISL induced apoptosis and invasion inhibition on breast cancer cells.

## Discussion

MiR-374a was first characterized as an oncogene in primary small cell lung cancer (NSCLC), and miR-374a overexpression could promote cell migration and invasion, positively correlating to the poor disease-free survival in NSCLC^10, 36^. The oncogenic effect of miR-374a on tumor invasion and metastasis contributed to its identification as cancerogenesis promoter in breast cancer progression by activating WNT/β-catenin signaling^12^. Recently, miR-374a has emerged as one of the major proliferative factors in carcinogenetic signaling. High expression of miR-374a promoted cell proliferation *in vitro* and tumor growth *in vivo* in in gastric cancer by targeting SRCIN1^37^. Overexpressed miR-374a in osteosarcoma remarkably accelerated cell proliferation by directly targeting AXIN2 and FOXO1, and silencing miR-374a could induce G_0_/G_1_ and G_1_/S arrest in cell cycle^38, 39^, suggesting its role as a potential therapeutic target. Based on our study, we also found high expression of miR-374a in breast cancer cells and tumors from breast cancer patients. However, the effect of miR-374a on breast cancer tumorigenesis was still unclear. Thus, we conducted a legitimate study to explore the functional ability of miR-374a in breast cancer growth. Using microarray screening, we demonstrated that miR-374a was one of the major miRNAs down-regulated by ISL treatment. The results were confirmed by RT-qPCR in a dose-dependent manner in both breast cancer cell lines and primary culture of breast cancer (see Supplementary Fig. S6). The decrease of miR-374a by ISL interference was in accordance with the inhibition of breast cancer growth and invasion. Also, our data supported that overexpressing miR-374a could at least partly reverse the inhibitory effect of ISL on cell mobile ability and the pro-apoptotic effect on cell dearth. Hence, our study clarified for the first time that natural compound ISL could induce apoptosis and inhibit metastasis by down-regulating miR-374a.

Akt pathway promotes cell survival as one of the major anti-apoptotic factors through the block of extracellular signal induced apoptosis^40^. Likewise, Akt signaling serves as a key activator of cell migration and invasion by phosphorylating a range of intracellular proteins^41^. Also, overexpression of pAkt was negatively associated with overall survival and disease-free survival of breast cancer patients^42^. Recently, ISL was reported as a promising inhibitor on Akt signaling in breast cancer^29, 30^. Therefore, we designed rationale experiments to investigate the underlying mechanisms involved Akt signaling pathway regulated by ISL. We collected ISL-treated MCF-7 and MDA-MB-231 cells and examined the phosphorylation of Akt through western blot analysis. Based on the results, Akt inhibition by ISL was in consistent with miR-374a down-regulation by ISL, indicating the role of miR-374a as one of the up-streams regulating Akt pathway. Our data manifested that ISL could promote PTEN expression *in vitro* and *in vivo*. Further study displayed that miR-374a could negatively regulate PTEN, the tumor suppressor of Akt signaling, through the direct binding with 3′ UTR of PTEN mRNA. Whether miR-374a can post-transcriptionally modulate PTEN needs to be further studied. The PTEN down-regulation by miR-374a and up-regulation by ISL were also confirmed through RT-qPCR and western blotting. Our findings indicated that ISL could down-regulate miR-374a, the negative regulator of PTEN, to suppress the significant oncogenic Akt signaling pathway.

Although our pervious study demonstrated that ISL and its derivatives inhibited breast cancer proliferation significantly^43^, studies examining the anti-cancer effect on mitochondrial-based apoptosis involving the release of Cyt C are very limited. Our data showed that ISL could dose-dependently increase the ratio of apoptotic cells after 24 h treatment. Interestingly, early apoptosis accounted for the majority of MCF-7 apoptotic cells, while ISL could induce a dramatic late apoptosis in MDA-MB-231, implying that MDA-MB-231 was more sensitive to response to ISL administration. Whether ISL could serve as a novel anti-cancer drug candidate with a more effective inhibitory effect on TNBCs needs to be further investigated. The western blot analysis exhibited that ISL could enlarge the percentage of released Cyt C in the cytoplasm with an increase of cleaved caspase-9 expression. The activated Akt could directly suppress proteolytic activity of caspase-9 via phosphorylating the protein^44^. These findings indicated that ISL could inactivate Akt pathway to enhance the activity of caspase-9, the positive regulator of Cyt C release in intrinsic apoptosis pathway.

In summary, our study exerted that ISL, a natural flavonoid, is a potent inhibitor of miR-374a in breast cancer. ISL inhibited cell proliferation and migration by down-regulating miR-374a expression, which negatively regulated PTEN, resulting in the induction of apoptosis and invasive inhibition through the inactivation of Akt pathway. These data provide novel insights into the function of miR-374a regulating breast cancer tumorigenesis, and further suggest a potential application of ISL as a miR-374a naturally inhibition candidate in breast cancer therapy.

## Materials and Methods

### Chemicals and Reagents

ISL was purchased from Alpha Aesar (MA, USA). Bovine serum albumin (BSA) Eosin Y, and Hematoxylin were purchased from Sigma (St. Louis, MO). All other reagents were obtained from standard commercial sources.

### Cell culture

MDA-MB-231, MCF-7, BT474, 4T1, MCF-10A and 293 T were obtained from the American Type Culture Collection (ATCC, USA) and maintained in a humidified incubator with 5% CO_2_ at 37 °C. MDA-MB-231, BT474 and 293 T were cultured in high glucose DMEM media, while MCF-7 and 4T1 were cultured in RPMI 1640 media, supplemented with 10% FBS and 1% penicillin and streptomycin (Gibco, Life Technologies, Lofer, AU). Spontaneously immortalized MCF10-A was cultured in keratinocyte serum-free medium (Gibco, Life Technologies, USA). Primary breast cancer was isolated from the distant metastatic position of a Luminal A breast cancer patient (Ethic approval obtained from Ethics Committee of Sun Yat-sen University Cancer Center, YB2016-002-03). The written informed consent was obtained. The corresponding experimental protocols were performed in accordance with guidelines of the Sun Yat-sen University Cancer Center (Guangzhou, China).

### Cell viability analysis

5 × 10^3^ cells/well were seeded in 96-well plates and incubated in presence of ISL at different dosages for 24 h, 48 h and 72 h. After treatment, 10 μl CCK-8 reagent (Biotool, Selleck Chemicals, Houston, USA) was added to the test well with 100ul cultured media and went through 3 h incubation in the incubator. Cell viability was recorded according to the absorbance at 450 nm by an ELISA plate reader. Triplicate experiments were performed independently.

### Flow cytometry analysis

Apoptotic cell percentages were determined by FITC-AnnexinV Apoptosis detection kit (BD Pharmingen, San Diego, CA, USA) as the manufacturer’s instructions. In brief, 5 × 10^5^ cells/well were seeded in 6-well plates and incubated with ISL at the indicated concentrations for 24 h. After harvested, cells were resuspend into 1X Binding Buffer at a concentration of 1 × 10^6^ cells/ml, and stained with FITC-conjugated Annexin V and PI in the dark. Triplicate experiments were performed independently. Stained samples were quantified by FACSAria SORP (BD Biosciences, San Jose, CA), and analyzed by FlowJo Software.

### TUNEL assay

Cell apoptosis dearth ratio was determined by TUNEL Apo-Green detection kit (Biotool, Selleck Chemicals, Houston, USA) as the manufacturer’s protocol. Briefly, monolayer cells were culture on the glass coverslips and, after the proper treatment, cells were fixed with 4% paraformaldehyde. Samples were permeabilizd in 0.2% Triton X-100 solution and stained with reaction mixture. Glass coverslips with cells were mounted with ProLong Gold Antifade Reagent with DAPI (Cell Signaling Technology, Danvers, MA). Triplicate experiments were performed independently. The images were detected by Axio Scope and analyzed by AxioVision Microscope 4.8 Software (Carl Zeiss AG, Jena, DE).

### Wound migration assay

3 × 10^5^ cells/ml were plated into Culture-Insert (ibidi GmbH, Martinsried, DE) in 70 μl cell suspensions. After cell attachment, Culture-Insert was gently removed and cells were treated with different reagents for another 24 h. Images were taken before the treatment and after the treatment. The migratory inhibitory effect of ISL was determined by measuring the closure of the wound between cells. Triplicate experiments were performed independently. The images were detected and analyzed by EVOS XL Core Imaging System (Invitrogen, Life Technologies, USA).

### Chamber migration and invasion assays

Cell migratory ability was assessed by 6-well transwell chambers (Corning Inc., Coring, USA) with 8-μm pore size. Briefly, 3 × 10^5^ cells/well were seeded on the upper side with different concentrations of ISL and lower chamber was filled with media (10% FBS). After 24 h, migrated cells were stained by 0.5% crystal violet. Cell invasive ability was assessed by 6-well matrigel-coated transwell chambers (Corning Inc., Coring, USA) with 8-μm pore size as similar previous description. Triplicate experiments were performed independently. The images were detected and analyzed by EVOS XL Core Imaging System (Invitrogen, Life Technologies, USA).

### Western blottin

Total protein was extracted using RIPA lysis (Sigma, St. Louis, MO). Mitochondrial and cytosolic proteins were collected using Mitochondria/Cytosol Fractionation Kit (EMD Millipore, Massachusetts, USA) referring to manufacturer’s protocol. Cell lysates (20 μg) fractionated electrophoretically by 12% SDS-PAGE gels, and transferred onto PVDF membranes (GE Healthcare, Freiburg, DE). Primary antibodies against Bax, Bcl-2, Caspase-9, Cyt C, PTEN, p-Akt (Ser473), Akt, p-GSK3β (Ser9), GSK3β, β-catenin and β-actin (Cell Signaling Technology, Danvers, MA) were probed with proteins on the membrane at 4 °C overnight. After incubating with secondary antibodies (Cell Signaling Technology, Danvers, MA), the results were detected by ECL Advance reagent (GE Healthcare, Freiburg, DE) with ChemiDoc XRS System and analyzed by Image Lab Software (Bio-Rad, Kidlington, UK).

### Immunohistochemistry analysis

Tumors were harvested from MMTV-PyMT transgenic mice at the end point of treatment. Paraffinized tumor sections were permeabilized in 0.2% Triton X-100 after de-paraffinized in xylene and rehydrated in ethanol. Tumor sections were immersed in sodium-citrate buffer (10 mM, pH 6.0) for antigen retrieval and blocked in 5% normal goat serum (Cell Signaling Technology, Danvers, MA). The slides were then incubated with diluted primary antibodies Bax, MMP-7, PTEN, and β-catenin (Cell Signaling Technology, Danvers, MA) at 4 °C overnight. After washing, the slides were incubated with SignalStain Boost Detection Reagent (Cell Signaling Technology, Danvers, MA) and developed color according to SignalStain DAB Substrate Kit (Cell Signaling Technology, Danvers, MA) manufacturer’s guidelines. The study was conducted in accordance with the guidelines approved by Department of Health (Hong Kong, Ethic approval, 15–322). The images were detected by Leica DME microscope (Meyer Instruments, Inc., Houston, USA) and analyzed by ScopeImage 9.0 Image-Processing Software (BP Integrated Technologies, Inc., Calamba, PH).

### Microarray analysis

Total RNA was extracted from 5 × 10^6^ cells of MDA-MB-231 and MCF-7 cells with or without 24 h ISL treatment using RNAiso Plus reagent (TaKaRa Bio Inc., Shiga, JP). Microarray analysis was performed with the Affymetrix GeneChip Human Gene 2.0 ST array (Affymetrix, Santa Clara, CA) by the Centre for Genomic Sciences of HKU. The results were analyzed by 7.0 Partek Genomics Suite 6.6 software (Partek Incorporated, Missouri, USA).

### Real-time RT–PCR

MiRNA was prepared using *mir*Vana miRNA Isolation Kit (Ambion, Life Technologies, USA) and total RNA was prepared with RNAiso Plus reagent based on the manufacturer’s protocol. For miRNA reverse transcription, cDNA was synthesized using miRCURY LNA microRNA cDNA synthesis kit II (Exiqon, Vedbaek, DK). For mRNA reverse transcription, cDNA was synthesized using PrimeScript RT Reagent Kit with gDNA Eraser (TaKaRa Bio Inc., Shiga, JP). Real-time PCR for miRNA and RNA were performed using ExiLENT SYBR Green master mix (Exiqon, Vedbaek, DK) and SsoFast EvaGreen Supermixes (Bio-Rad, Kidlington, UK), respectively. Relative quantification was determined by normalization to GAPDH or U6. The primers are shown in Table S1.

### *In situ* hybridization (ISH) analysis

Expression of miR-374a in formalin-fixed paraffin-embedded archive tissues tissues and normal tissues from 39 breast cancer patients (TMA) (US Biomax, Inc., Rockville, USA) collected in 2004 was determined by ISH with probes for miR-374a. Each tissue spot was accompanied with cases material including sex, age, pathologic type, pathologic grade and clinical stage. The staining intensity was defined as described previously^45^. The study was conducted in accordance with the guidelines of Ethics Committee of Sun Yat-sen University Cancer Center (Guangzhou, China) and Tongxuxian People’s Hospital (Henan, China) (Ethic approval, 081116). A written informed consent was obtained from all participants involved in this study. The corresponding experimental protocols were approved by Ethics Committee of Sun Yat-sen University Cancer Center (Guangzhou, China). The images were detected by B203LED microscopy (Optec, Chongqing, CN) and analyzed by Image J (National Institutes of Health, Maryland, USA).

### Cell transfection

Diluted RNA oligonucleotides of *mir*Vana miRNA-374a mimic and *mir*Vana miRNA Mimic Negative Control (Ambion, Life Technologies, USA) were mixed with diluted Lipofectamine RNAiMAX Transfection Reagent (Invitrogen, Life Technologies, USA) as 1:1 ratio and incubated for 5 minutes at room temperature. The miRNA-lipid complex was then added to cells for 24 h transfection. Diluted RNA oligonucleotides of *Silencer* Select Negative Control PTEN siRNA and *Silence*r Select Negative Control siRNA (#4390843, Ambion, Life Technologies, USA) were mixed with diluted Lipofectamine 2000 Transfection Reagent (Invitrogen, Life Technologies, USA) as 1:1 ratio and incubated for 15 minutes at room temperature. The siRNA-lipid complex was then added to cells for 48 h transfection. The oligo sequences of siRNA PTEN are shown in Table S2.

### Luciferase reporter assay

The amplified PTEN wide-type/mutant fragments were cloned into the Sac I and HindIII restriction sites of the pMIR-REPORT Luciferase plasmid (Ambion, Life Technologies, USA) using In-Fusion Dry-Down PCR Cloning Kit (TaKaRa Bio Inc., Shiga, JP). The primers are shown in Table S3. 293 T cells were seeded into 96-well plates with transfection mixture. The luciferase activity was recorded using the Dual-Glo Luciferase Assay System (Promega, Madison, WI) by normalization to Renilla luciferase activity according to manufacturer’s instructions.

### Statistical analysis

All the data were expressed as means ± standard deviations (SD). Two-tailed student’s *t* test, one way ANOVA, and Chi-square test were used to determine the significant difference of different experiment results by 13.0 SPSS (SPSS Inc., Chicago, USA) software. Survival curves were plotted using the Kaplan–Meier method. Data with *P* < 0.05 were considered as statistical significance.

### Data availability

The datasets analyzed in Supplementary Fig. S3 during the current study are available in the array express repository, (https://www.ebi.ac.uk/arrayexpress/).

## Electronic supplementary material

Supplementary Information
