# Author Correction: Isoliquiritigenin modulates miR-374a/PTEN/Akt axis to suppress breast cancer tumorigenesis and metastasis

**DOI:** 10.1038/s41598-023-36401-z

**Published:** 2023-06-20

**Authors:** Fu Peng, Hailin Tang, Peng Liu, Jiangang Shen, Xinyuan Guan, Xiaofang Xie, Jihai Gao, Liang Xiong, Lei Jia, Jianping Chen, Cheng Peng

**Affiliations:** 1 School of Chinese Medicine, The University of Hong Kong, Pokfulam, Hong Kong; 2 Chengdu University of Traditional Chinese Medicine, Chengdu, China; 3 State Key Laboratory Breeding Base of Systematic Research, Development and Utilization of Chinese Medicine Resources, Sichuan Province and Ministry of Science and Technology, Chengdu, China; 4 Department of Breast Oncology, Sun Yat-Sen University Cancer Center; State Key Laboratory of Oncology in South China; Collaborative Innovation Center of Cancer Medicine, Guangzhou, China; 5 Department of Clinical Oncology, Li Ka Shing Faculty of Medicine, the University of Hong Kong, Pokfulam, Hong Kong

Correction to: *Scientific Reports* 10.1038/s41598-017-08422-y, published online 21 August 2017

This article contains an error in Figure 5.

As a result of an error during figure assembly, images collected for the same sample were used to represent different conditions in Figure 5A, timepoint 0h. The corrected Figure 5 and its accompanying legend appear below.

**Figure 5:**
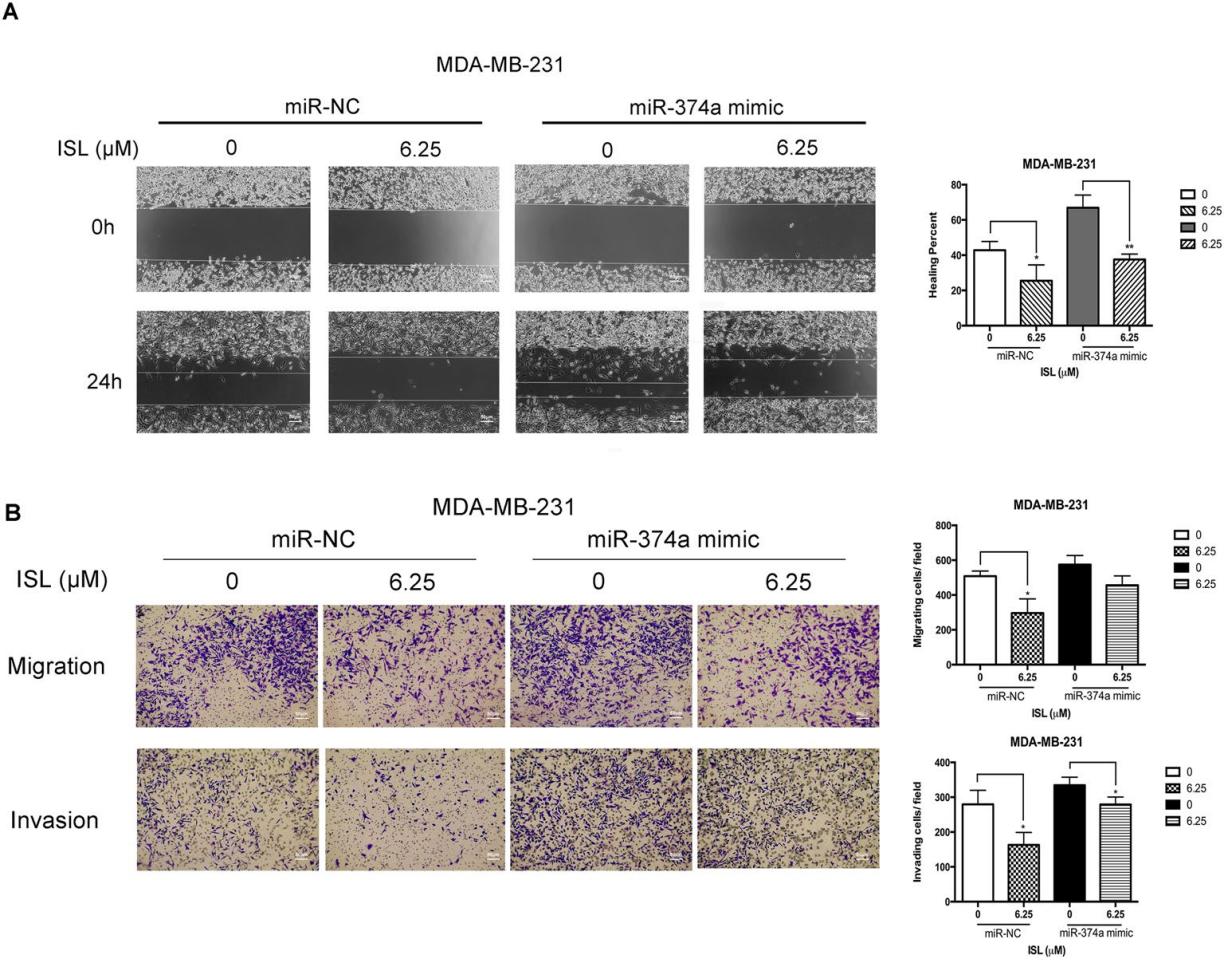
Pretreatment of miR-374a in MDA-MB-231 attenuates the responses to ISL. (**A**) Representative images of wound healing assay in miR-NC and miR-374a mimic transfected groups after 24 h ISL treatment. (**B**) Chamber migration and invasion assay analysis of the effect of miR-374a transfection on breast cancer motile ability with ISL interference. (**C**) Percentages of closures of the wound in miR-374a-modulated MDA-MB-231 cells by 24 h exposure to ISL. (**D**,**E**) Percentages of the number of cells located on the lower side of chambers and Matrigel-coated chambers in the presence of ISL with miR-374a interference. Data represent the mean ± s.d. *P < 0.05, **P < 0.01.

