# Supplementary material for: Isoliquiritigenin modulates miR-374a/PTEN/Akt axis to suppress breast cancer tumorigenesis and metastasis
Source: Sci Rep. 2017 Aug 21;7:9022. doi: 10.1038/s41598-017-08422-y (PMC5567123; doi:10.1038/s41598-017-08422-y)
Supplement: Supplementary file 1 — Supplementary Information [file 41598_2017_8422_MOESM1_ESM.pdf]

SUPPLEMENTAL INFORMATION

Supplementary Figure 1-10

Supplementary Table 1-3

Isoliquiritigenin modulates miR-374a/PTEN/Akt axis to suppress breast cancer tumorigenesis and metastasis

Fu Peng[1,2,3#], Hailin Tang[1,4#], Peng Liu[4], Jiangang Shen[1], Xinyuan Guan[5], Xiaofang Xie, [2,3] Jihai Gao[2,3], Liang Xiong[2,3], Lei Jia[1], Jianping Chen[1,2*], & Cheng Peng[2,3*]

[1]School of Chinese Medicine, The University of Hong Kong, Pokfulam, Hong Kong
[2]Chengdu University of Traditional Chinese Medicine, Chengdu, China
[3]State Key Laboratory Breeding Base of Systematic Research, Development and Utilization of Chinese Medicine Resources, Sichuan Province and Ministry of Science and Technology, Chengdu, China
[4]Department of Breast Oncology, Sun Yat-sen University Cancer Center; State Key Laboratory of Oncology in South China; Collaborative Innovation Center of Cancer Medicine, Guangzhou, China
[5]Department of Clinical Oncology, Li Ka Shing Faculty of Medicine, the University of Hong Kong, Hong Kong

[*] **Corresponding authour**: Jianping Chen, School of Chinese Medicine, The University of Hong Kong, 10 Sassoon Road, Pokfulam, Hong Kong. E-mail: abchen@hku.hk；Cheng Peng, Chengdu University of Traditional Chinese Medicine, Chengdu, China. Email: pengchengchengdu@126.com
[#] Two authors equally contributed to this work.

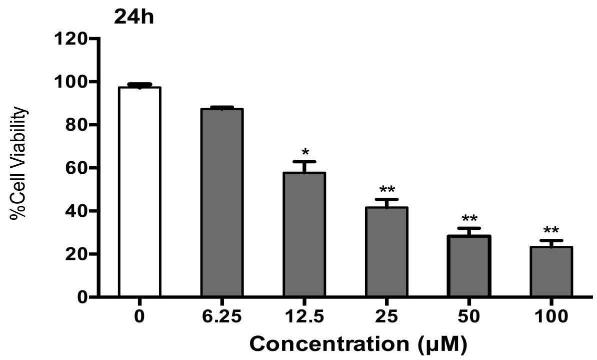

Supplementary Figure 1. MTT analysis demonstrated that ISL had an inhibitory effect on primary breast cancer proliferation in a dose-dependent manner. MTT was used to determine the anti-proliferation effect of ISL on primary breast cancer after 24h treatment.

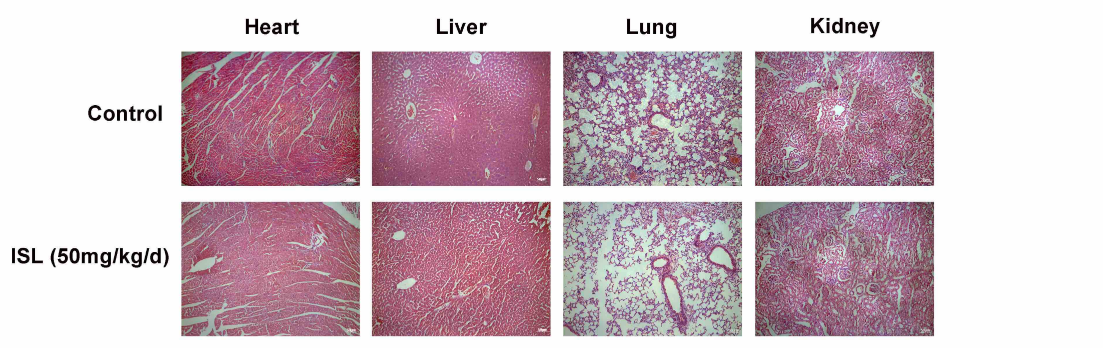

Supplementary Figure 2. HE analysis demonstrated that ISL had little influences on the micro-morphology of normal tissues. HE staining was used to determine the effect of ISL on the heart, liver, lung and kidney.

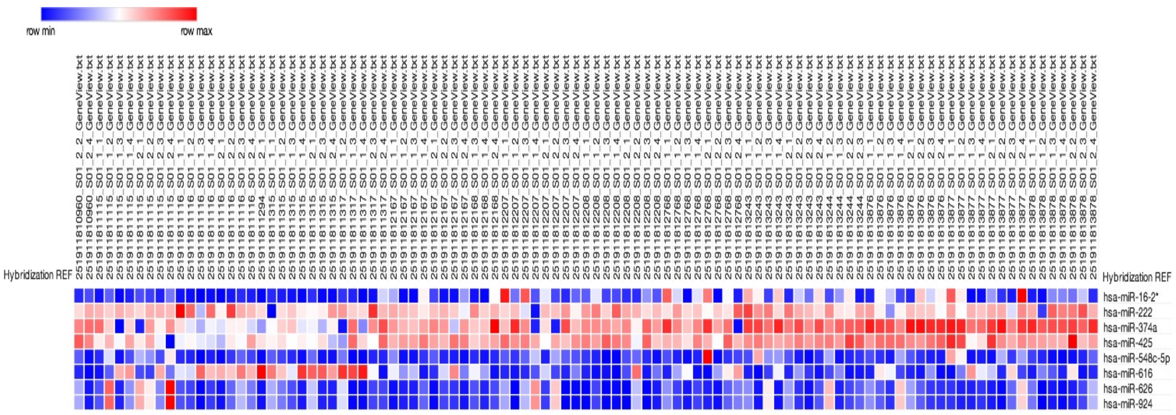

Supplementary Figure 3. MiR-374a was highly expressed in breast cancer tumors compared to non-tumor tissues. The data from array express (https://www.ebi.ac.uk/arrayexpress/) was determined by online analysis (https://software.broadinstitute.org/morpheus/) to detect miRNAs expression down-regulated by ISL in breast cancer samples.

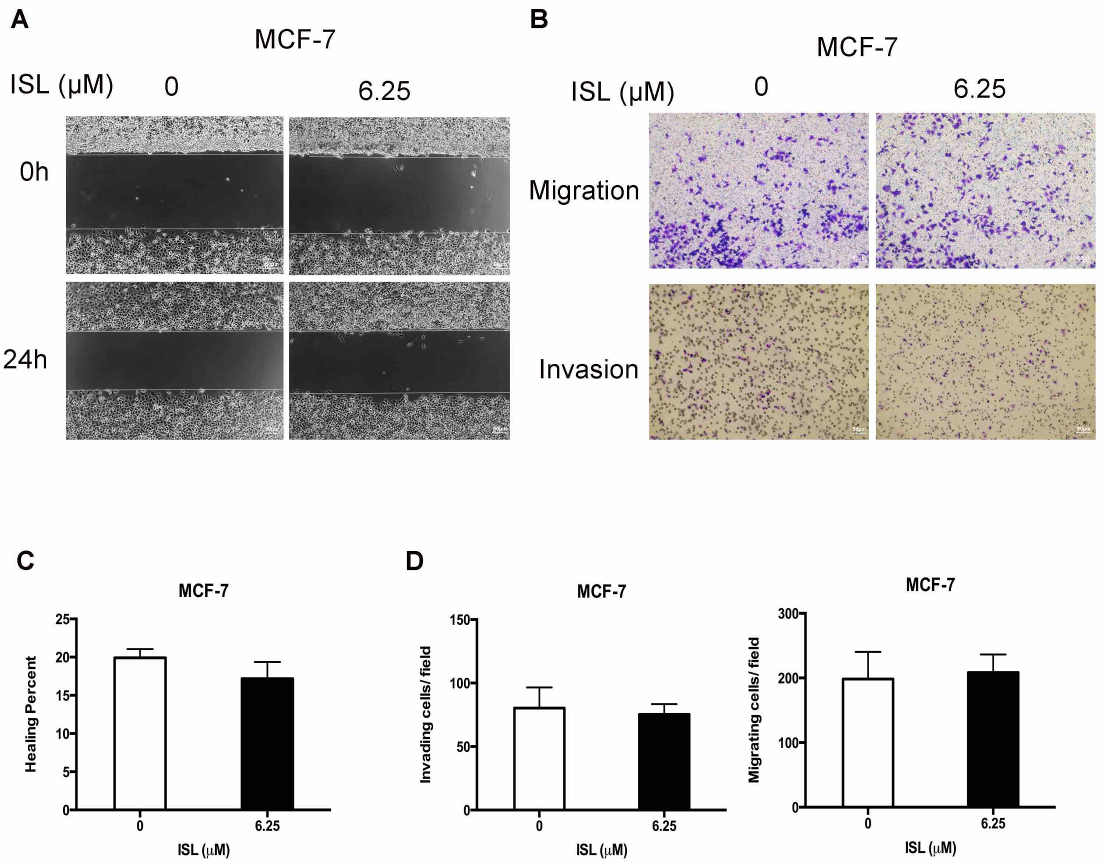

Supplementary Figure 4. ISL demonstrated no obvious inhibitory effect on MCF-7 migration and invasion. (A, C) The effect of ISL on MCF-7 migration was determined by wound healing assay. (B, D) The effect of ISL on MCF-7 migration and invasion was determined by chamber assay.

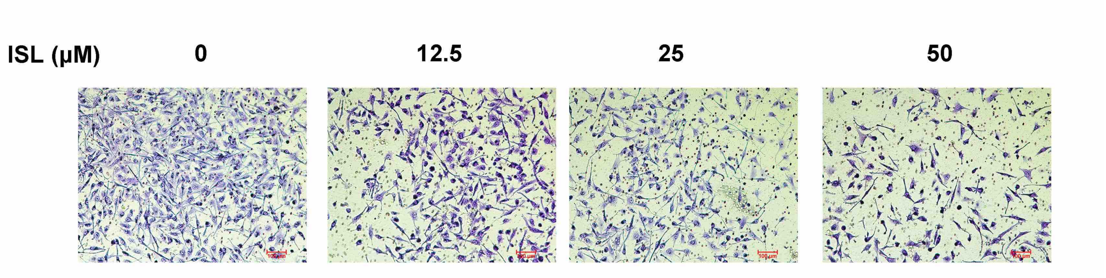

Supplementary Figure 5. ISL demonstrated an inhibitory effect on primary breast cancer invasion in a dose-dependent manner. The effect of ISL on primary breast cancer invasion was determined by chamber assay.

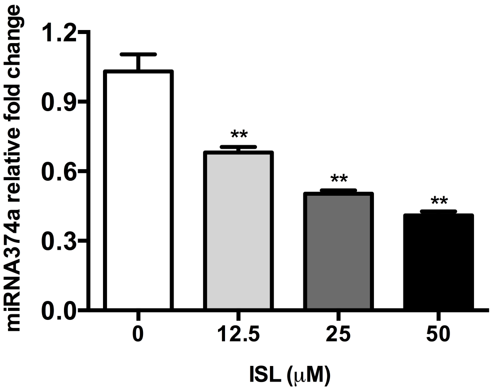

Supplementary Figure 6. Real-time PCR analysis of primary breast cancer indicated that ISL could inhibit miR-374a expression dose-dependently. Real-time PCR was used to determine the effect of ISL on primary breast cancer miR-374a expression with 24h intervention.

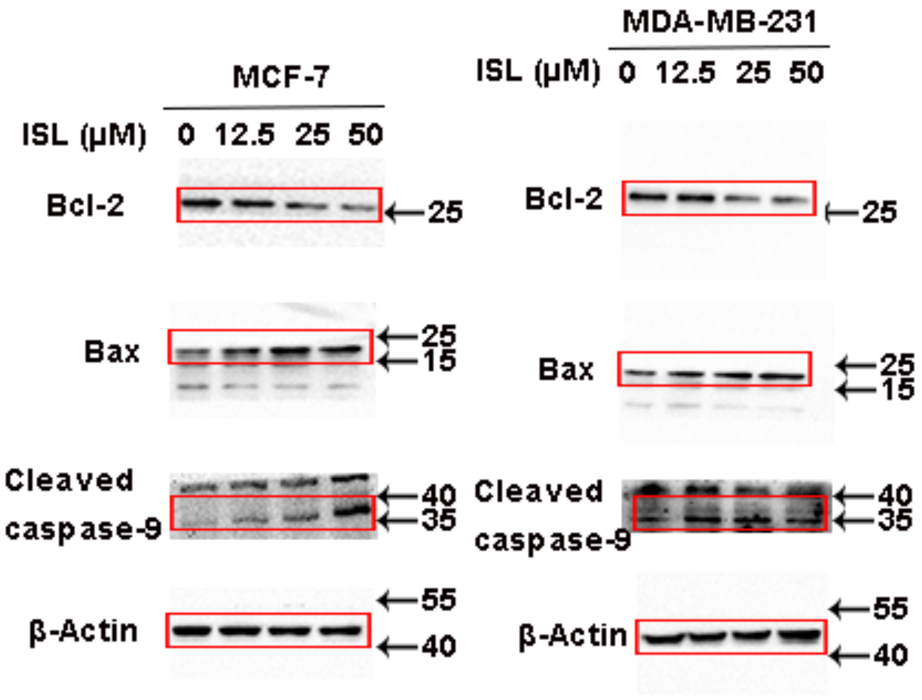

Supplementary Figure 7. Full-length images of the immunoblots. Red line boxes indicate the cropped images used in Figure 1D. β-actin was used as an internal control. The same set of protein samples was charged in all gels. Arrowheads indicate the position of protein markers.

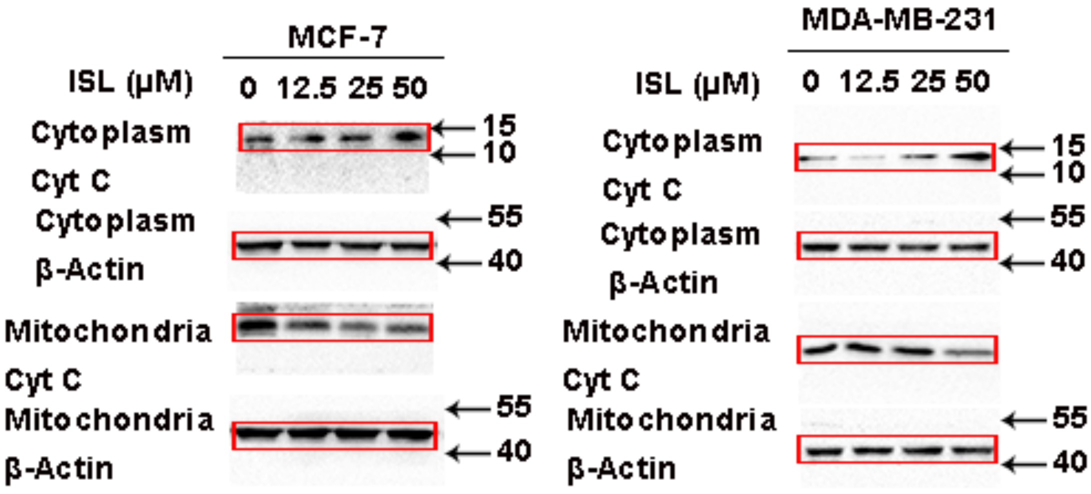

Supplementary Figure 8. Full-length images of the immunoblots. Red line boxes indicate the cropped images used in Figure 1E. β-actin was used as an internal control. The same set of protein samples was charged in all gels. Arrowheads indicate the position of protein markers.

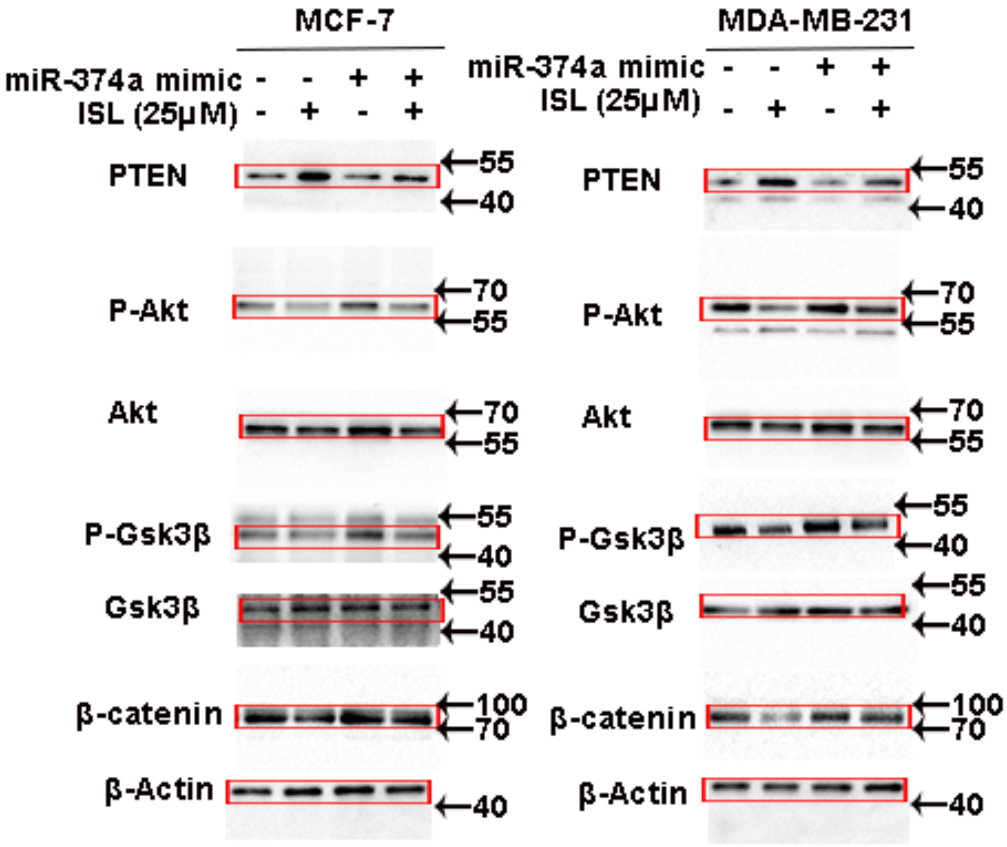

Supplementary Figure 9. Full-length images of the immunoblots. Red line boxes indicate the cropped images used in Figure 6D. β-actin was used as an internal control. The same set of protein samples was charged in all gels. Arrowheads indicate the position of protein markers.

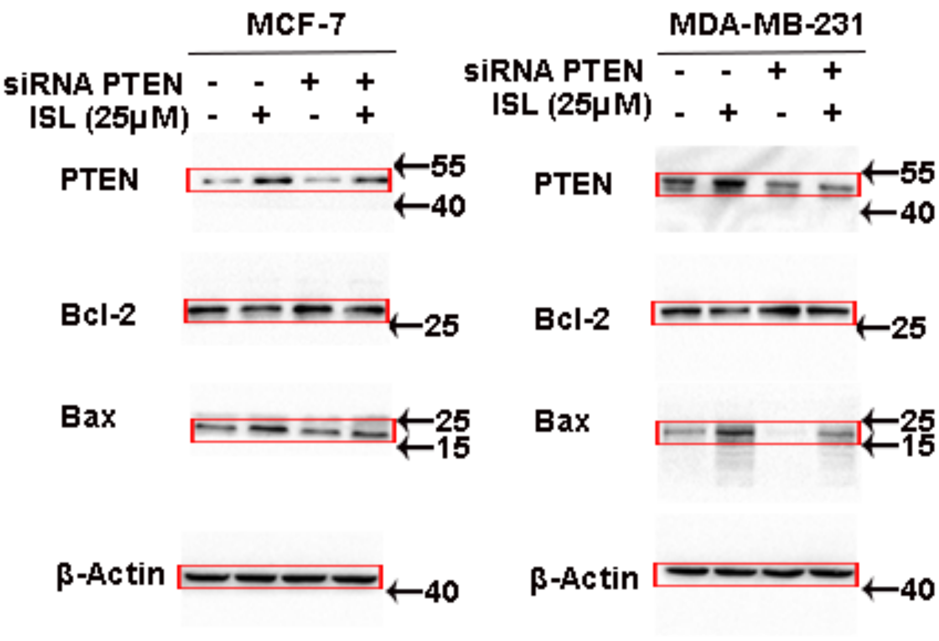

Supplementary Figure 10. Full-length images of the immunoblots. Red line boxes indicate the cropped images used in Figure 7G. β-actin was used as an internal control. The same set of protein samples was charged in all gels. Arrowheads indicate the position of protein markers.

Table S1. The primers used for Real-time RT–PCR

**PTEN**	(F) AACGCTTCACGAATTTGCGT
	(R) AGAGGAGCAGCCGCAGAAATG
**BAX**	(F) CCGCCGTGGACACAGACT
	(R) TTGAAGTTGCCGTCAGAAAACA
**BCL-2**	(F) CTGGGAATCGATCTGGAAATCC
	(R) TGCATAAGGCAACGATCCCATC
**GAPDH**	(F) GACTCATGACCACAGTCCATGC
	(R) AGAGGCAGGGATGATGTTCTG
**U6**	(F) CTCGCTTCGGCAGCACA
	(R) AACGCTTCACGAATTTGCGT

Table S2. The oligo sequences of siRNA PTEN

**siPTEN**	(Sense) GGUUUUCGAGUCCUAAUUAtt
	(Antisense) UAAUUAGGACUCGAAAACCtt

Table S3. The primers used for colony PCR

**PTEN wide-type**	(F) ATGAGCTCATGTGAAGGTCTGAATGAGG
	(R) GCAAGCTTTTCAAGAGGAGCTACAAAGG
**PTEN mut-1**	(F) GGAAAAATGGCATTATATATGTTGTGTATATAAATATATATTATA
	(R) TATAATATATATTTATATACACAACATATATAATGCCATTTTTCC
**PTEN mut-2**	(F) TATTATATATATAAATATATGTTGTGCATACTCTCCTTACTTTAT
	(R) ATAAAGTAAGGAGAGTATGCACAACATATATTTATATATATAATA
